# Malignancies After Heart Transplantation

**DOI:** 10.3389/ti.2024.12109

**Published:** 2024-09-09

**Authors:** Caroline Stenman, Andreas Wallinder, Erik Holmberg, Kristjan Karason, Jesper Magnusson, Göran Dellgren

**Affiliations:** ^1^ Transplant Institute, Sahlgrenska University Hospital, Gothenburg, Sweden; ^2^ Department of Cardiothoracic Surgery, Sahlgrenska University Hospital, Gothenburg, Sweden; ^3^ Regional Cancer Center West, Region Västra Götaland, Sahlgrenska Academy, University of Gothenburg, Gothenburg, Sweden; ^4^ Department of Oncology, Institute of Clinical Sciences, Sahlgrenska Academy, University of Gothenburg, Gothenburg, Sweden; ^5^ Department of Cardiology, Sahlgrenska University Hospital, Gothenburg, Sweden; ^6^ Department of Molecular and Clinical Medicine, Institute of Medicine, Sahlgrenska Academy, University of Gothenburg, Gothenburg, Sweden; ^7^ Department of Internal Medicine/Respiratory Medicine and Allergology, Sahlgrenska University Hospital, Gothenburg, Sweden

**Keywords:** cancer, heart transplantation, epidemiology, cohort study, single center study

## Abstract

Heart transplant patients have an increased risk of developing cancer. Patients who underwent HTx between 1985 and 2017 were included. Detection of cancer was obtained by cross-checking the study population with the Swedish Cancer-Registry and the Cause-of-Death-Registry. A total of 664 patients were followed for a median of 7.7 years. In all, 231 malignancies were diagnosed in 138 patients. Compared to the general population the excess risk of cancer following HTx was 6.2-fold calculated as the standardized incidence ratio (SIR) and 2.9-fold after exclusion of non-melanoma skin cancer (NMSC). The most common malignancies were NMSC, non-Hodgins lymphoma, and lung cancer. There was no significant difference in overall survival between those with and without a history of cancer before HTx (*p* = 0.53). During a median follow-up of 7.7 years, 19% of HTx recipients developed cancer, 6.2-fold higher relative to the general population, and 2.9-fold higher when excluding NMSC. Risk factors for malignancies (excluding NMSC) included previous smoking, hypertension and prolonged ischemic time; and for NMSC, increasing age, seronegative CMV-donors, and azathioprine. A previous cancer in selected recipients results in similar survival compared to those without cancer prior to HTx.

## Graphical Abstract

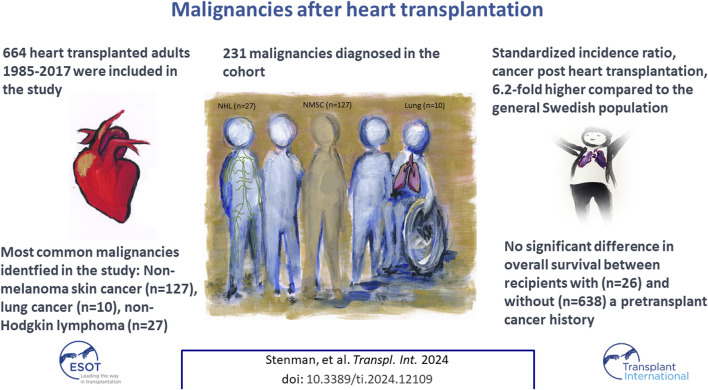

## Introduction

Heart transplantation (HTx) is a life-saving treatment for end-stage heart failure. Long-term survival after HTx is limited by several risk factors.; one of them being malignancies. As compared with the general population, solid organ recipients have a 2-4-fold higher risk of developing cancer [1–3], a major cause of morbidity and mortality [4].

Compared with recipients of abdominal organs, thoracic transplant recipients have a higher risk of developing cancer. The leading cause is the higher doses of immunosuppressive agents needed to prevent organ rejection [5–7]. The most frequent cancers after HTx have been reported to be non-melanoma skin cancer (NMSC), lung cancer and lymphomas [8].

The present study examines the incidence of post-transplant cancer and the survival rate among HTx recipients diagnosed with cancer at the Sahlgrenska University Hospital (SUH). Survival rates are compared with the general population in Sweden. It was also studied if a history of treated cancer before HTx affected post-transplant cancer incidence and survival.

## Patients and Methods

**FIGURE 1:**
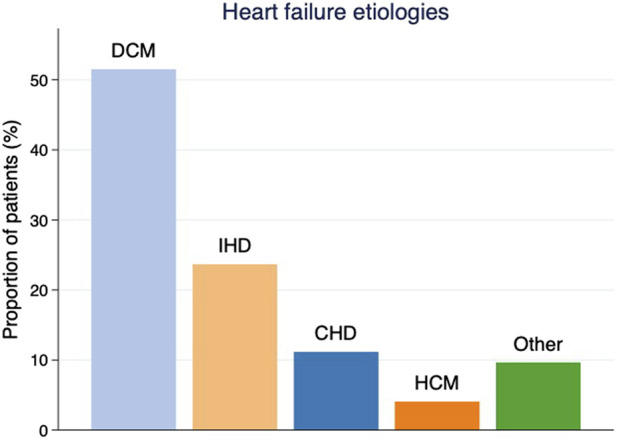
Etiology of heart failure as proportions of patients by diagnosis prior to heart transplantation. DCM = dilated cardiomyopathy, IHD = ischemic heart disease, CHD = congenital heart disease, HCM = hypertrophic cardiomyopathy.

Between June 1985 and December 2017, 708 HTx were performed at the SUH. After exclusion of patients treated with re-transplantation (n = 21) and patients followed abroad (n = 23), a total of 664 patients were included in the study. Baseline characteristics are shown in Table 1. The mean age was 48 years and 74% of the cohort were men**.** The median follow-up time was 7.7 years generating a total of 5,668 patient-years. Figure 1 depicts the etiology of heart failure in the study population. The study was conducted in accordance with the Declaration of Helsinki and was approved by the Regional Ethical Review Board at University of Gothenburg (EPN no. 019-09, approval date 22nd October 2009, amendments approved 29th November 2010, 10th December 2012, 17th December 2013, 10th May 2017). The main outcome was the detection of cancer among participants, by cross-checking the study cohort with the Swedish Cancer Register (SCR) [9] and the Swedish Cause of Death Register (SCDR), which both are operated by the Swedish National Board of Health and Welfare. The following cancer diagnosis codes were used: 140-239 (International Classification of Diseases-9 until 1996) and codes C00-D48 (International Classification of Diseases-10 from 1997). Data on basal cell carcinoma (BCC) were not available from these registers, and are, therefore, not included in our NMSC population.

**TABLE 1:** Patient characteristics*.

Characteristics	
Age, years	48 (33–57)
Follow-up time, years	7.7 (3.0–13.6)
Gender	
Males	494 (74)
Females	170 (26)
Smoking	
Previous, cessation <6 months before HTx	57 (9)
Previous, cessation >6 months before HTx	227 (34)
Never	355 (53)
Missing data	25 (4)
Previous malignancy, and type	
Acute lymphoblastic leukemia (ALL)	3 (11.1)
Adrenal gland	1 (3.7)
Breast	2 (7.4)
Corpus uteri	1 (3.7)
Hodgkin lymphoma	3 (7.4)
Connective and soft tissue of lower limb including hip	1 (3.7)
Connective and soft tissue of thorax	1 (3.7)
Lymphosarcoma	3 (11.1)
Ovary	1 (3.7)
Other primary malignant neoplasm of lymphoid tissue	1 (3.7)
Other skin of other and unspecified parts of face	1 (3.7)
Pelvic bones, sacrum and coccyx	1 (3.7)
Prostate	1 (3.7)
Stomach	2 (3.7)
Testis	1 (3.7)
Unspecified axilla and upper limb lymph nodes	1 (3.7)
Unspecified part of bronchus or lung	1 (3.7)
Vertebral column	1 (3.7)
Total	26 (100)
Donors	
Males	405 (61%)
Females	255 (38%)
Missing data	4 (1%)
Median age of the donor, years	40.0 (23–51)

*Values are presented as medians with interquartile ranges or numbers and percentages within parenthesis.

All Swedish citizens have a specific personal identity number that is registered at all healthcare contacts. This allows tracking of how the Swedish population interacts with the healthcare system [10]. The SCR, that originates from 1958 holds information on all citizens registered in Sweden with a cancer diagnosis. The SCDR, which stems from 1961, registers the cause of death, including cancer, for all deceased citizens registered in Sweden. All cancer diagnoses in this report were verified by a histopathological examination. The International Rules for Multiple Primary Cancers (IARC, ICD-0 Third Edition) were applied to ensure correct numbers of cancer tumors reported [11]. These rules enable comparison of cancer risk and outcome between different populations.

Listing criteria for HTx were coherent with established international guidelines [12]. Recipients and donors were matched for ABO blood group compatibility. Complement-dependent cytotoxicity assays were performed to assess the recipient serum’s ability to lyse a panel of T or B cells and, if positive above a certain level, a prospective donor-specific cross-match was performed. At the beginning of our HTx program, bi-atrial technique was initially used but this was slowly changed to bi-caval technique during the period as formerly described [13]. Over time, the complexity of surgery has increased, and the number of patients bridged with mechanical circulatory support has risen. Survival has improved over time, which has previously been reported [14].

Induction therapy has been applied throughout our HTx program (n = 573), mainly anti-thymocyte globulin. All patients received a regime with three different immunosuppressive agents, including a calcineurin inhibitor (cyclosporine or tacrolimus), an antimetabolite (azathioprine or mycophenolate mofetil (MMF) and a corticosteroid, tapered during the first year. Previously, cyclosporine and azathioprine constituted the primary immunosuppressive treatment but were replaced with tacrolimus and MMF during the 2000s. According to a routine protocol, percutaneous transvenous myocardial biopsies were used for rejection monitoring. All patients received acetylsalicylic acid and a statin as a preventive measure for graft vasculopathy.

Data are presented as means and standard deviations, medians and interquartile ranges, or numbers and percentages. Overall survival curves were generated using Kaplan-Meier estimates and comparisons between groups were performed with the log rank test. Relative survival was calculated using the Ederer II method [15]. Mortality data for the general population in Sweden were used to estimate expected survival rates. The mortality data comprised the probability of death for single-year age groups in 1-year calendar period. Cumulative incidence of cancer was analyzed using competing risk methods with death as a competing event [16]. When analyzing cancer incidence for different cancer types, person-years were calculated from date of the transplantation to the first of the following events: diagnosis of the cancer site; death; or end of surveillance period, i.e., 31 December 2018 The standardized incidence ratio (SIR) was defined as the observed number of cancers during the observation time divided by the expected number of cases, using incidence rates from the Swedish population stratified for 5-year age groups (0–4, 5–9, … 80–84, 85-), gender and calendar year. Incidence rates for different cancer sites were used from the NORDCAN project. Furthermore, the coding of cancer followed definitions according to International rules for multiple primary cancers [11]. Univariable and multivariable risk factor analyses by Cox proportional hazards regression model for the development of posttransplant malignancy were performed. The following parameters were tested by univariable analyses: age (per 10 years), sex, BMI (<20; 20–30; >30), smoking (never; cessation >6 months before HTx listing; cessation <6 months before HTx listing), hypertension, diabetes mellitus, TIA/stroke, previous cardiac surgery, donor age (per 10 years), CMV+/− donor, CMV+/− recipients, CMV mismatch, ventricular assist device (VAD), ischemic time (<3; 3–4; >4 h), total induction dose with ATG (<200; 200–800; >800 mg) and proliferation inhibitors (azathioprine vs. MMF). Significant risk factors in the univariate models for all cancers and NMSC, respectively (Supplementary Tables S1, S2) were tested also in a multivariable model. All statistical tests were two-sided, and a *p*-value of <0.05 was considered statistically significant. Statistical analyses were carried out with Stata/IC 16.1.

## Results

Mortality for the whole patient cohort within 30 days and 1-year was 51/664 (8%) and 78/664 (12%), respectively. Overall survival was for the whole cohort at 1, 5, 10, 15 and 20 years was: 88% (95% CI 86%–90%); 80% (95% CI 76%–83%); 67% (95% CI 63%–71%); 53% (95% CI 48%–58%) and 37% (95% CI 32%–43%), respectively. Overall survival for HTx patients increased significantly over time (*p* < 0.001). Five-year overall survival for those who underwent HTx between 1985 and 2000 was: 70% (95% CI: 64%–75%); between 2001 and 2010 81% (95% CI: 75%–86%); and between 2011 and 2017 92% (95% CI: 85%–96%) (Supplementary Figure S1).

A total of 231 *de novo* cancers were diagnosed in 138 HTx patients during follow-up, which corresponds to 19.6% of the total study population (Table 2). In contrast 37.5 detected cancers would have been in a cohort from the general population matched by age, sex and time period. This resulted in a SIR of 6.2 (95% CI 5.4–7.0) for all cancers and a SIR of 2.9 (95% CI: 2.4–3.5) after exclusion of NMSC.

**TABLE 2:** Number of cancers after HTx according to ICD-10.

Site (ICD-10)	Sex		Total
	Males	Females	
Acute myeloblastic leukemia (AML) (C92)		1	1
Anus (C21)	1		1
Bladder (C67)	1		1
Breast (C50)		3	3
Brain (C71)	2		2
Colon (C18)	1		1
Cerebral meninges (C70)	2	1	3
Cervix uteri (C53)		1	1
Connective and soft tissue (C49)	1		1
Extrahepatic bile duct (C24)	1		1
Gastric (C16)	4	1	5
Glottis (C32)	1	1	2
Hodgkin lymphoma (C81)		1	1
Kidney (C64)	3		3
Lip (C00)	7		7
Lung (C34)	8	2	10
Lymph nodes (C77)	1		1
Malignant melanoma (C43)	4	2	6
Malignant of other and ill-defined sites (C76)	1		1
Multiple Myeloma (C90)	4		4
Non-Hodgkin lymphoma (C83-C85)	22	5	27
Nasal cavity (C30)	1		1
Penis (C60)	2		2
Prostate (C61)	12		12
Oral cavity (C03,C08)	2		2
Other malignant neoplasms of skin (C44)	110	17	127
Tonsil (C09)	1		1
Upper respiratory tract (C39)	1		1
Urethra (C66)	1		1
Vulva (C51)		2	2
Total	195	36	231

The cumulative incidence of a *de novo* malignancy between 1 and 5 years after transplant was 4.6%. The cumulative incidence of cancer for the total cohort at 1, 5, 10, 15 and 20 years was 2.4% (95% CI 1.5–3.9), 7.0% (95% CI 5.2–9.3), 19% (95% CI 15.8–23), 26% (95% CI 22–31) and 33% (95% CI 28–38), respectively.

**FIGURE 2:**
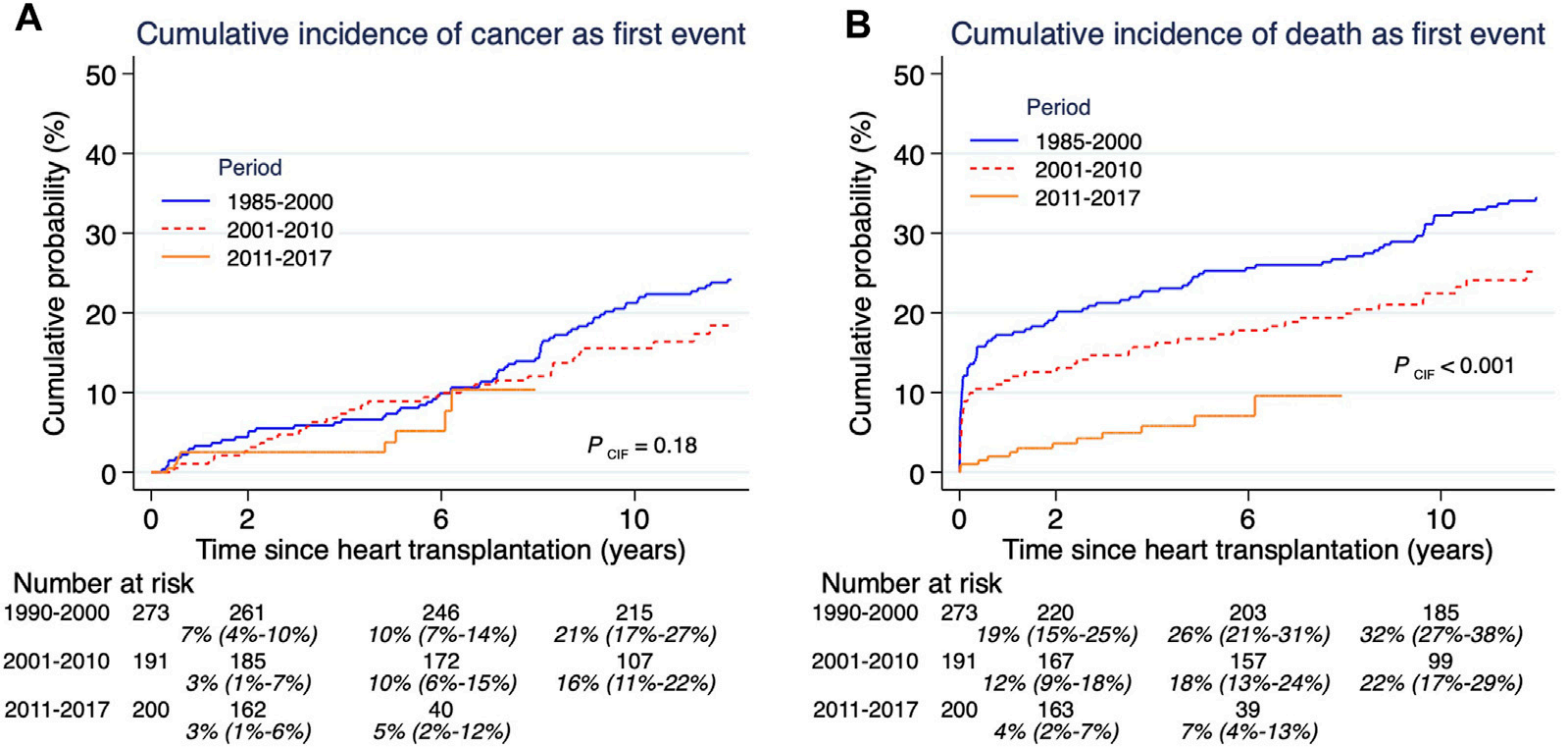
Cumulative incidence of competing risks cancer **(A)** and death **(B)**, and related to time-era after heart transplantation. Both outcomes cancer and death need to be assessed together since they are competing outcomes. Estimates and 95% confidence intervals are shown under the number of patients at risk.

Cumulative incidence of post-HTx cancers by three prespecified time periods showed no difference over time (Figure 2A). During these same eras there was a significant decrease in overall cumulative mortality (Figure 2B).

The type and frequency of solid tumors for the total group, and for men and women separately, are listed in Table 2. The most common type of cancer was NMSC (55% of all cancers), non-Hodgin’s lymphoma (11.7% of all cancers) and lung cancer (4.3% of all cancers).

Among the patients who developed lung cancer (eight men and two women), 80% had a smoking history. Among them, four patients had stopped smoking <6 months before HTx listing and four had stopped >6 months before HTx listing.

A total of 44 patients (32%) had multiple tumors; 18 patients had 2 tumors, 6 patients had 3 tumors, 3 patients had 4 tumors and 5 patients had 5 tumors. Two patients developed 7 tumors each. One had 5 NMSC, one lip tumor and one salivary gland tumor, and the other had 5 NMSC, 1 anal cancer and 1 salivary gland tumor.

The SIR for cancer types diagnosed in four persons or more, are shown in Table 3. The excess risk of all cancers for the total population was 6.2 and similar between men and women (SIR 6.39 and SIR 5.18, respectively).

**TABLE 3:** Observed and expected cancer risks following HTx.*

Site (ICDO-10)	Observed number	Expected number	Person years	SIR (95% CI)
All sites Total Males Females	231 195 36	37.5 30.5 6.95	5,668 4,175 1,493	6.16 (5.42–7.01) 6.39 (5.55–7.35) 5.18 (3.74–7.18)
All sites (except NMSC) Total Males Females	104 85 19	35.9 29.2 6.73	5,668 4,175 1,493	2.89 (2.39–3.51) 2.91 (2.35–3.60) 2.82 (1.80–4.43)
Lip (C00) Total Males Females	7 7 0	0.10 0.08 0.01	5,668 4,175 1,493	72.6 (34.6–152) 83.0 (39.6–174) —
Stomach (C16) Total Males Females	5 4 1	0.64 0.56 0.08	5,668 4,175 1,493	7.86 (3.27–18.9) 7.13 (2.68–19.0) 13.3 (1.87–94.5)
Breast (C32) Total Males Females	3 0 3	2.38 0.05 2.33	5,668 4,175 1,493	1.26 (0.41–3.91) 1.28 (0.41–3.98)
Lung (C33, C34) Total Males Females	10 8 2	2.72 2.21 0.51	5,668 4,175 1,493	3.68 (1.98–6.84) 3.62 (1.81–7.25) 3.93 (0.98–15.7)
Malignant melanoma (C43) Total Males Females	6 4 2	0.64 0.56 0.08	5,668 4,175 1,493	9.43 (4.24–21.0) 7.13 (2.68–19.0) 26.6 (6.66–106)
Skin, NMSC (C44) Total Males Females	127 110 17	1.54 1.31 0.23	5,668 4,175 1,493	82.6 (69.4–98.3) 84.0 (69.7–101) 74.7 (46.4–120)
Prostate (C61) Males	12	10.6	4,110	1.13 (0.64–1.99)
Kidney (C64) Total Males Females	3 3 0	0.94 0.82 0.12	5,668 4,175 1,493	3.20 (1.03–9.92 3.65 (1.18–11.3)
Non-Hodgkin lymphoma (C81-C85) Total Males Females	27 22 5	1.09 0.92 0.17	5,668 4,175 1,493	24.8 (17.0–36.1) 23.9 (15.7–36.3) 29.4 (12.2–70.6)
Myeloma (C90) Total Males Females	4 4 0	0.44 0.38 0.06	5,628 4,135 1,493	9.08 (3.41–24.2) 10.6 (3.99–28.3)

*Observed and expected number of cancers, person years in follow-up and Standardized Mortality Ratio (SIR) per site after heart transplantation. NMSC, non-melanoma skin cancer (not including basal cancer).

The overall incidences of cancers in the cohort were higher than expected for: NMSC (SIR 82.6, 95% CI 69.4–98.3); non-Hodgkin lymphoma (SIR 24.8, 95% CI 17–36.1); malignant melanoma (SIR 9.42, 95% CI 4.24–21.0); multiple myeloma (SIR 9.08, 95% CI 3.41–24.2); gastric (SIR 7.86, 95% CI 3.27–18.9); lung (SIR 3.68, 95% CI 1.98–6.84); and kidney (SIR 3.20, 95% CI 1.03–9.92).

Among the 138 HTx patients who developed post-HTx cancer, 29 died from their malignancy: non-Hodgkin lymphoma (n = 13), lung cancer (n = 8), NMSC (n = 5), and gastric (n = 3).

Univariable and multivariable associations between baseline factors and cancer (omitting NMSC) are shown in Table 4. Independent predictors of cancer development included: smoking cession <6 months before HTx listing [HR 3.46 (95% CI 1.69–7.07), *p* < 0.001]; hypertension [HR 2.16 (95% CI 1.10–4.26), *p* < 0.026]; ischemic time (reference: <3 h) 3–4 h [HR 1.93 (95% CI 1.09–3.40), *p* = 0.024]; and treatment with azathioprine (vs. MMF) [HR 1.69 (95% CI 0.99–2.90), *p* < 0.055]. Interestingly, CMV mismatch was not significantly associated with cancer [HR 0.79 (95% CI 0.39–1.62), *p* = 0.53].

**TABLE 4:** Uni- and multivariable Cox proportional hazard regression for developing cancer.*

Risk factors for any cancer except skin cancer after HTx					
Variable	Number of persons with cancer/N	Cox proportional hazard regression			
		Univariable		Multivariable	
		HR (95% CI)	*p*	HR (95% CI)	*p*
Smoking					
No, never	23/355	1.0 (ref.)		1.0 (ref.)	
No, stopped >6 months before HTx	27/227	1.84 (1.05–3.21)	0.032	1.70 (0.96–3.02)	0.070
No, stopped < 6 months before HTx	12/57	3.12 (1.55–6.27	0.001	3.46 (1.69–7.07)	0.001
Hypertension					
No	51/565	1.0 (ref.)		1.0 (ref.)	
Yes	11/74	1.92 (1.00–3.68)	0.050	2.16 (1.10–4.26)	0.026
Ischemic time (hours)					
<3	23/285	1.0 (ref.)		1.0 (ref.)	
3–4	30/274	1.44 (0.84–2.48)	0.19	1.93 (1.09–3.40)	0.024
>4	10/97	1.59 (0.75–3.34)	0.22	1.92 (0.87–4.24)	0.11
Proliferation inhibitors					
MMF	23/355	1.0 (ref.)		1.0 (ref.)	
Azathioprine	40/288	1.68 (1.00–2.83)	0.050	1.69 (0.99–2.90)	0.055
Risk factors for skin cancer after HTx					
Age, per 10 years	40/664	2.53 (1.77–3.62)	<0.001	2.90 (1.85–4.55)	<0.001
Hypertension					
No	29/565	1.0 (ref.)		1.0 (ref.)	
Yes	11/74	3.74 (1.86–7.49)	<0.001	1.87 (0.91–3.84)	0.091
CMV donor					
Positive	19/400	1.0 (ref.)		1.0 (ref.)	
Negative	18/205	2.01 (1.05–3.83)	0.034	2.14 (1.11–4.14)	0.024
Proliferation inhibitors					
MMF	13/355	1.0 (ref.)		1.0 (ref.)	
Azathioprine	27/288	1.54 (0.79–3.01)	0.20	2.53 (1.20–5.36)	0.015

HTx, heart transplantation; CMV, cytomegalo virus; MMF, mycophenolate mofetil.

*Time to first cancer analyzed. Twenty years follow-up.

Significant risk factors in the multivariable model predicting NMSC only (Table 4) were: age per 10 years [HR 2.90 (95% CI 1.85–4.55), *p* < 0.001]; hypertension [HR 1.87 (95% CI 0.91–3.84), *p* < 0.091], seronegative CMV-donor [HR 2.14 (95% CI 1.11–4.14), *p* = 0.024], and treatment with Azathioprine [HR 2.53 (95% CI 1.20–5.36), *p* < 0.015].

**FIGURE 3:**
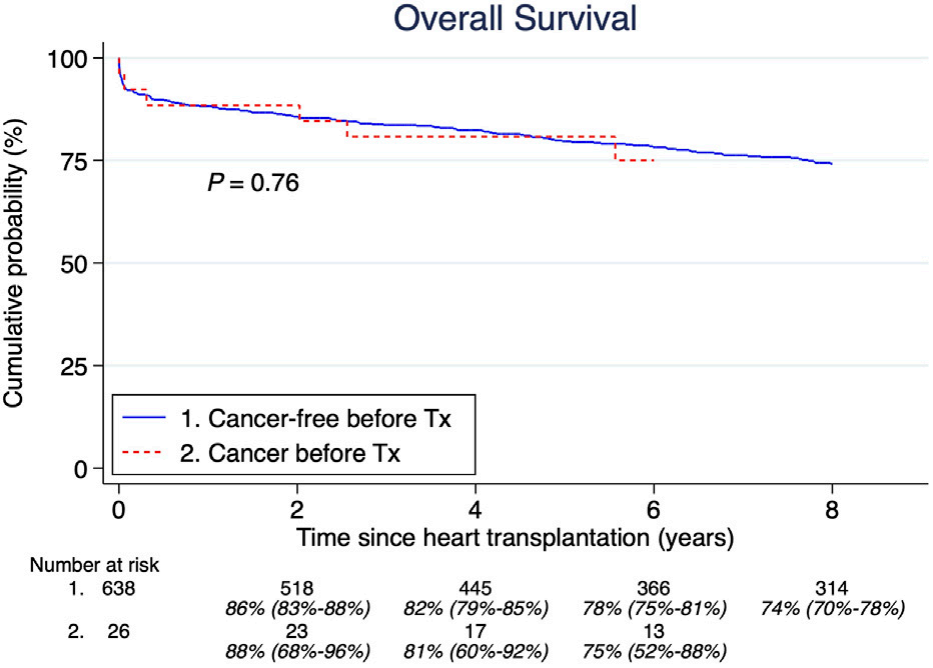
Pretransplant malignancy overall survival compared to those without. Estimates and 95% confidence intervals are shown under the number of patients at risk.

**FIGURE 4:**
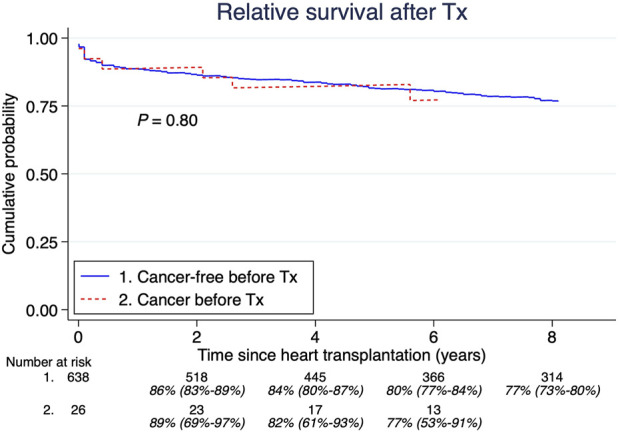
Pretransplant malignancy relative survival compared to those without. Estimates and 95% confidence intervals are shown under the number of patients at risk.

A total of 26 patients (4%) had a malignancy history >5 years before HTx. The median age at their first tumor was 34 years (IQR; 11.3–52.2 years). The most common cancers were: acute lymphoblastic leukemia, lymphosarcoma, and Hodgkin lymphoma, shown in Table 1. There were no significant differences in overall survival between those with and without cancer before HTx (Figure 3). Furthermore, there was no significant difference in post-HTx relative survival between patients who were cancer-free before and after HTx (Figure 4).

## Discussion

In the current study, we observed a 6.2-fold excess risk of cancer following HTx relative to the general population. When excluding NMSC, the excess risk was 2.9-fold. The cumulative incidence of a *de novo* malignancy between 1 and 5 years after HTx was 4.6%, which is lower compared to previous studies. In a study by Yoan et al., including over 17,000 patients [17] the corresponding incidence rate of *de novo* malignancy was 10.7%, which is considerably higher than the 4.6% rate observed in the present study. Yoan et al., derived data from the ISHLT registry, with a high rate of missing data supported by the fact that 24,000 HTx patients were excluded from the analyses. We therefore argue that they overestimated the risk of cancer due to positive selection. In contrast, we have derived data from national full coverage registries with a very low risk of missing any cancer. Data extraction from complete registries with detailed information on individual level makes this study unique. An improved post-HTx survival was observed during later eras, this could not be explained by a reduction in cancer incidence, which remained stable over the study period. This could indicate earlier detection and/or improved post-HTx cancer treatment.

It is well known that transplanted patients, as compared to the general population, have a higher incidence of NMSC, and the detected tumors are more aggressive [18]. It is also acknowledged that the incidence of NMSC increases with the intensity and duration of immunosuppression [19]. The SIR for NMSC of 82.6 in our cohort was more than four times higher as compared to the SIR of 18.5 reported by Collett et al. [3]. However, Collett et al. noted that their results might have been underestimated, since lesions might have been removed without a histological examination. A study from Finland, including 479 patients reported a SIR of 51.9 for squamous cell carcinoma (SCC) [20], which is also lower than we observed. Crespo Leiro et al. [21] and Vaan Keer et al. [22] studied the incidence of NCSC in a Spanish and Belgian population, but didn´t compare the cancer incidence to the general population, why SIR could not be calculated. In our study, almost 55% of the tumors (127 of 231) were NMSC. Corresponding numbers in the studies by Crespo-Leiro et al. and Vann Keer et al. were 51% (324 of 490) and 22% (58 of 263). The median duration of patient follow-up was 7.7 years in the present study compared to with 5.8 years in the study by Crespo-Leiro et al. and 10.7 years in the study by Vaan Keer et al.

While lung cancer in men has declined in Sweden since the 1980s, the opposite trend has been observed in women, to the point where more women than men now develop lung cancers [23]. The overall SIR for lung cancer (3.68) was higher than observed in other comparable studies (2.0–2.79) [3, 24–27]. There was no significant increased risk for lung cancer in transplanted women presented in a Spanish study by Crespo-Lieiro et al. [25], with over four thousand patients. Among patients with a history of smoking in our study, 284 patients (43%) had stopped smoking 6 months or more before being listed for transplantation. Crespo-Lieiro et al [25] showed an association between previous smoking and development of post-HTx lung cancer. In that study the median time between HTx and the lung cancer diagnosis was 6.4 years, compared to 7.8 years (IQR: 4.1–12.2) in our study. In addition to hypertension and longer ischemic time, previous smoking was also a risk factors for the development of NMSC in the present study.

The SIR for prostate cancer in our cohort was low (1.13), indicating that immunosuppression after HTx was not associated with a significantly increased prostate cancer risk. Similar findings have also been reported in a meta-analysis including six independent studies, with over 21,000 heart transplant patients [28]. Why immunosuppression does not increase the risk for prostate cancer remains largely unknown, but this may in part be explained by intensive screening before HTx, and the possibility that male HTX recipients don’t survive long enough to develop prostate cancer, which mostly occurs after the age of 70 years [23]. These findings support that patients with low-grade prostate cancer could be accepted for HTx without the often applied 5-year cancer free waiting period.

An excess risk of lip cancer was demonstrated (SIR 72.6) corroborating findings by Collet et al (SIR 60) and Jääma-Holmberg et al (SIR 47.4). The reason remains unclear, but may be related to the fact that this is a NMSC, and therefore can be classified as skin cancer. There is an association between smoking and lip cancer and the fact that two patients diagnosed with lip cancer (seven lip cancers among four persons) had a history of smoking may partially explain our findings. Sun exposure is also a well-established risk factor for the development of lip cancer [29]. Among the seven patients with a lip cancer, five (71%) also had a NMSC.

A history of malignancy within 5 years has been considered to be a contraindication for transplantation because of the increased risk of recurrence following initiation of immunosuppressive therapy. Sigurdardottir et al. [30] reported a cancer recurrence rate of 63%, if the disease-free interval between malignancy and HTx was less than 1 year, but only 6% if the HTx recipient had been cancer free for more than 5 years. However, prostate cancer was not presented as a separate entity, why the post-HTx relapse of prostate cancer remains unclear. In a review article by Mistiaen et al [31], patients with localized prostate cancer before transplantation had no increased risk of reduced survival post-HTx [31]. At present, there is no consensus regarding the optimal time interval between cancer treatment and HTx [32, 33], but a relapse free period of ≥5 years is a common criterion, which has also been applied at our center. However, when post HTx survival is constantly improving, at the same time as cancer incidence remains unchanged, it might be time to revise and consider individualization of these criteria.

### Limitations

The current study did not investigate whether the patients had BCC before or after HTx, since this cancer form has been considered rather benign and, therefore, not reported in the SCR. The growth rate of BCC is slow and this cancer rarely metastasizes [34]. Post-transplant lymphoproliferative disease (PTLD), a well-known complication after HTx [35] is not recorded as a specific entity in the SCR, why the exact number of patients with PTLD is cannot be reported. Instead, such cases are presented as lymphomas.

There are multiple primary cancer coding rules to count incident cases. Cancer registries in the U.S. and Canada use, for example, SEER multiple primary rules. In this study, the International Rules for Multiple Primary Cancers (IARC/IACR, ICD-0 Third Edition) was applied. Compared to SEER rules, IARC/IACR recognizes fewer multiple primary cancers [36], which may explain the differences compared to other studies [26].

## Data Availability

The original contributions presented in the study are included in the article/Supplementary Material, further inquiries can be directed to the corresponding author.
