# Supplementary material for: Malignancies After Heart Transplantation
Source: Transpl Int. 2024 Sep 9;37:12109. doi: 10.3389/ti.2024.12109 (PMC11417470; doi:10.3389/ti.2024.12109)
Supplement: Supplementary file 1 [file Table1.pdf]

Supplementary Table 1.

Uni- and multivariable Cox proportional hazard regression for developing any cancer (excluding NMSC) after HTx.*

**Variable**	Number of persons with cancer/N	Cox proportional hazard regression			
		Univariable		Multivariable	
		HR (95% CI)	p	HR (95% CI)	p
Age, per 10 years	63/664	1.11 (0.96-1.30)	0.16		
Gender					
Male	49/494	1.0 (ref.)			
Female	14/170	0.79 (0.43-1.42)	0.43		
BMI					
<20	6/85	0.28 (0.28-1.51)	0.31		
20-30	48/449	1.0 (ref.)			
>30	3/77	0.25 (0.13-1.37)	0.15		
Smoking					
No	23/355	1.0 (ref.)		1.0 (ref.)	
No, ended >6 mon before HTx	27/227	1.84 (1.05-3.21)	0.032	1.70 (0.96-3.02)	0.070
No, ended <6 mon before HTx	12/57	3.12 (1.55-6.27	0.001	3.46 (1.69-7.07)	0.001
Hypertension					
No	51/565	1.0 (ref.)		1.0 (ref.)	
Yes	11/74	1.92 (1.00-3.68)	0.050	2.16 (1.10-4.26)	0.026
Diabetes					
No	60/591	1.0 (ref)			
Yes	3/68	0.47 (0.15-1.49)	0.20		
TIA/Stroke					
No	55/570	1.0 (ref.)			
Yes	6/79	0.76 (0.33-1.77)	0.53		

Previous heart surgery					
No	40/381	1.0 (ref)			
Yes	22/270	0.92 (0.54-1.54)	0.74		
Donor age, per 10 years	62/660	0.94 (0.81-1.09)	0.44		
CMV donor					
Negative	25/205	1.0 (ref.)			
Positive	33/400	0.66 (0.40-1.12)	0.12		
CMV recipient					
Negative	16/186	1.0 (ref.)			
Positive	46/463	1.03 (0.58-1.83)	0.91		
CMV donor/recipient					
Other combinations	49/480	1.0 /ref.)			
Donor pos / Recipient neg	9/121	0.79 (0.39-1.62)	0.53		
VAD					
No	56/522	1.0 (ref.)			
Yes	7/122	0.67 (0.31-1.48)	0.32		
Ischemic time (hours)					
<3	23/285	1.0 (ref.)		1.0 (ref.)	
3-4	30/274	1.44 (0.84-2.48)	0.19	1.93 (1.09-3.40)	0.024
>4	10/97	1.59 (0.75-3.34)	0.22	1.92 (0.87-4.24)	0.11
Induction, T-cell antibody (mg)					
<200	21/205	1.0 (ref.)			
200-800	28/370	0.63 (0.36-1.12)	0.11		
>800	12/57	1.71 (0.84-3.49)	0.14		
Proliferation inhibitors					
MMF	23/355	1.0 (ref.)		1.0 (ref.)	
Azathioprine	40/288	1.68 (1.00-2.83)	0.050	1.69 (0.99-2.90)	0.055

*Time to first cancer analyzed. Twenty years follow-up.
HTx = heart transplantation, NMSC = non-melanoma skin cancer, BMI = body mass index, TIA = transient ischemic attack, CMV = cytomegalo virus, VAD = ventricular assist device, MMF = mycophenolate mofetil
