# Supplementary material for: Malignancies After Heart Transplantation
Source: Transpl Int. 2024 Sep 9;37:12109. doi: 10.3389/ti.2024.12109 (PMC11417470; doi:10.3389/ti.2024.12109)
Supplement: Supplementary file 2 [file Table2.pdf]

Supplementary Table 2.

Uni- and multivariable Cox proportional hazard regression for developing any NMSC after HTx.*

Variable	Number of persons with cancer/N	Cox proportional hazard regression			
		Univariable		Multivariable	
		HR (95% CI)	p	HR (95% CI)	p
Age, per 10 years	40/664	2.53 (1.77-3.62)	<0.001	2.90 (1.85-4.55)	<0.001
Gender					
Male	35/494	1.0 (ref.)			
Female	5/170	0.39 (0.15-0.99)	0.048		
BMI					
<20	5/85	0.79 (0.31-2.01)	0.62		
20-30	32/449	1.0 (ref.)			
>30	3/77	0.71 (0.22-2.31)	0.57		
Smoking					
No	15/355	1.0 (ref.)			
No, ended >6 mon before HTx	21/227	1.97 (1.01-3.81)	0.046		
No, ended <6 mon before HTx	4/57	1.37 (0.46-4.14	0.57		
Hypertension					
No	29/565	1.0 (ref.)		1.0 (ref.)	
Yes	11/74	3.74 (1.86-7.49)	<0.001	1.87 (0.91-3.84)	0.091
Diabetes					
No	34/591	1.0 (ref)			
Yes	6/68	1.82 (0.76-4.33)	0.18		
Stroke					
No	34/570	1.0 (ref.)			
Yes	6/79	1.27 (0.53-3.03)	0.58		
Previous heart surgery					
No	30/381	1.0 (ref)			
Yes	10/270	0.56 (0.27-1.15)	0.11		
Donor age, per 10 years	40/660	1.14 (0.94-1.39)	0.18		
CMV donor					
Negative	18/205	1.0 (ref.)		1.0 (ref.)	
Positive	19/400	0.50 (0.26-0.95)	0.034	0.47 (0.24-0.90)	0.024
CMV recipient					
Negative	7/186	1.0 (ref.)			
Positive	33/463	1.63 (0.72-3.69)	0.24		
CMV donor/recipient					
Other combinations	32/480	1.0 /ref.)			
Donor pos / Recipient neg	5/121	0.68 (0.27-1.75)	0.43		

VAD					
No	38/522	1.0 (ref.)			
Yes	2/122	0.32 (0.08-1.31)	0.11		
Ischemic time (hours)					
<3	17/285	1.0 (ref.)			
3-4	21/274	1.37 (0.72-2.60)	0.33		
>4	2/97	1.59 (0.75-3.34)	0.22		
Induction, T-cell antibody (mg)					
<200	9/205	1.0 (ref.)			
200-800	23/370	1.09 (0.50-2.36)	0.82		
>800	7/57	2.04 (0.76-5.49)	0.16		
Proliferation inhibitors					
MMF	13/355	1.0 (ref.)		1.0 (ref.)	
Azathioprin	27/288	1.54 (0.79-3.01)	0.20	2.53 (1.20-5.36)	0.015

*Time to first cancer analyzed. Twenty years follow-up.
BMI = body mass index, NMSC = non-melanoma skin cancer, TIA = transient ischemic attack, CMV = cytomegalo virus, VAD = ventricular assist device, MMF = mycophenolate mofetil
