# Supplementary figures and images for: Malignancies After Heart Transplantation
Source: Transpl Int. 2024 Sep 9;37:12109. doi: 10.3389/ti.2024.12109 (PMC11417470; doi:10.3389/ti.2024.12109)

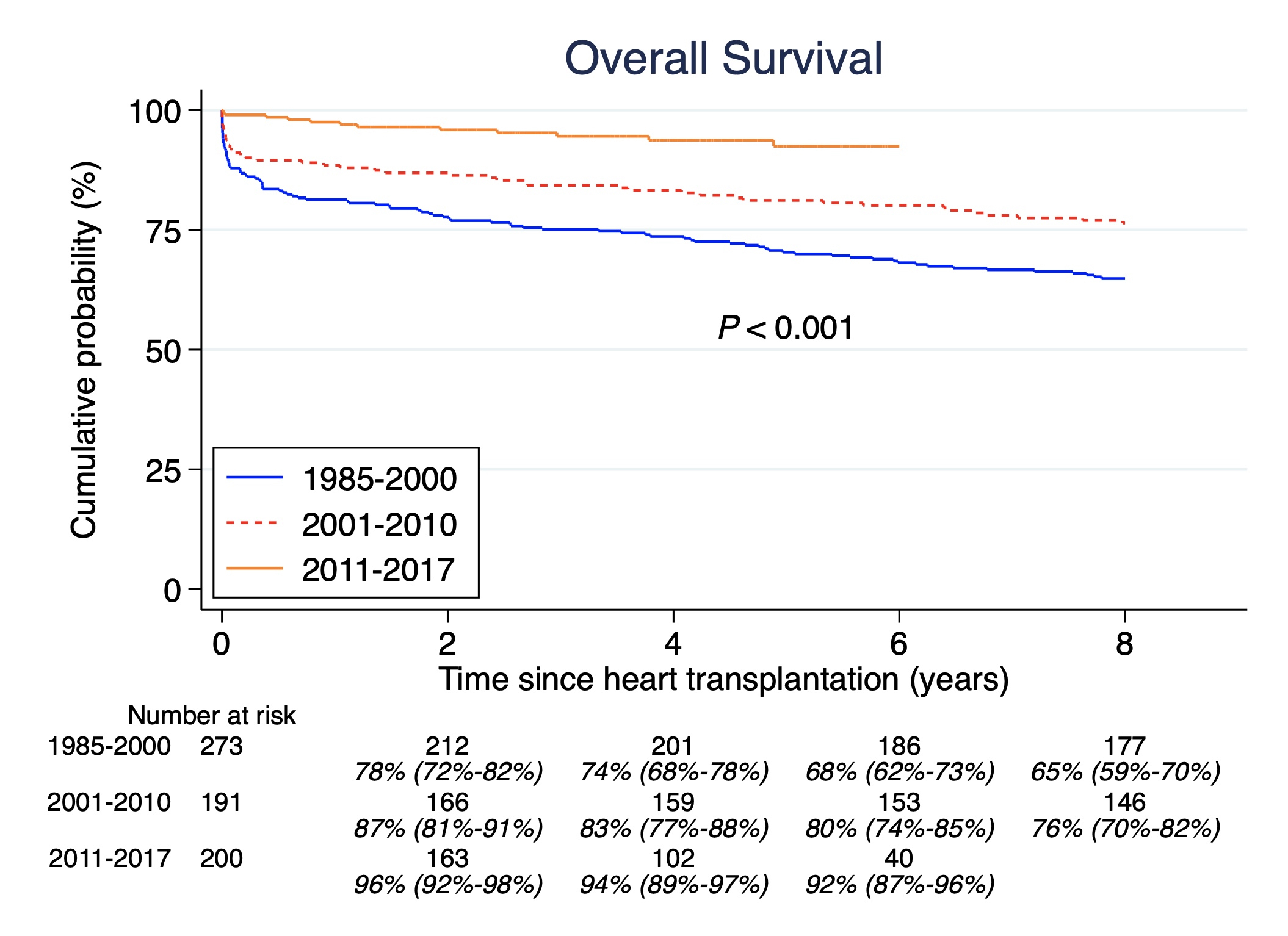

Supplement: Supplementary file 3 [file Image1.jpg]
